# Labor of Minors

**Published:** 1856-05

**Authors:** 


*Labor of Minors.*—A ten hour law was reported on the same day (March 21st,) in the House, from a joint special committee on the subject, which provides that after July 4th, no minor shall be required to work in incorporated establishments more than sixty hours per week, or an average of ten hours per day.—*Ibid.*

